# Comparisons of intensity-duration patterns of physical activity in the US, Jamaica and 3 African countries

**DOI:** 10.1186/1471-2458-14-882

**Published:** 2014-08-27

**Authors:** Lara R Dugas, Pascal Bovet, Terrence E Forrester, Estelle V Lambert, Jacob Plange-Rhule, Ramon A Durazo-Arvizu, David Shoham, Jacolene Kroff, Guichan Cao, Richard S Cooper, Soren Brage, Ulf Ekelund, Amy Luke

**Affiliations:** Stritch School of Medicine, Loyola University Chicago, Maywood, IL USA; Institute of Social & Preventive Medicine, Lausanne University Hospital, Lausanne, Switzerland; Ministry of Health, Victoria, Seychelles; Tropical Medicine Research Institute, University of the West Indies, Mona, Kingston, Jamaica; Research Unit for Exercise Science and Sports Medicine, University of Cape Town, Cape Town, South Africa; Kwame Nkrumah University of Science and Technology, Kumasi, Ghana; Medical Research Council Epidemiology Unit, University of Cambridge, Cambridge, UK; Department of Sports Medicine, Norwegian School of Sports Sciences, Oslo, Norway

**Keywords:** Physical activity patterns, Manual labor, Epidemiologic transition

## Abstract

**Background:**

This difference in how populations living in low-, middle or upper-income countries accumulate daily PA, i.e. patterns and intensity, is an important part in addressing the global PA movement. We sought to characterize objective PA in 2,500 participants spanning the epidemiologic transition. The Modeling the Epidemiologic Transition Study (METS) is a longitudinal study, in 5 countries. METS seeks to define the association between physical activity (PA), obesity and CVD risk in populations of African origin: Ghana (GH), South Africa (SA), Seychelles (SEY), Jamaica (JA) and the US (suburban Chicago).

**Methods:**

Baseline measurements of objective PA, SES, anthropometrics and body composition, were completed on 2,500 men and women, aged 25–45 years. Moderate and vigorous PA (MVPA, min/d) on week and weekend days was explored ecologically, by adiposity status and manual labor.

**Results:**

Among the men, obesity prevalence reflected the level of economic transition and was lowest in GH (1.7%) and SA (4.8%) and highest in the US (41%). SA (55%) and US (65%) women had the highest levels of obesity, compared to only 16% in GH. More men and women in developing countries engaged in manual labor and this was reflected by an almost doubling of measured MPVA among the men in GH (45 min/d) and SA (47 min/d) compared to only 28 min/d in the US. Women in GH (25 min/d), SA (21 min/d), JA (20 min/d) and SEY (20 min/d) accumulated significantly more MPVA than women in the US (14 min/d), yet this difference was not reflected by differences in BMI between SA, JA, SEY and US. Moderate PA constituted the bulk of the PA, with no study populations except SA men accumulating > 5 min/d of vigorous PA. Among the women, no sites accumulated >2 min/d of vigorous PA. Overweight/obese men were 22% less likely to engage in manual occupations.

**Conclusion:**

While there is some association for PA with obesity, this relationship is inconsistent across the epidemiologic transition and suggests that PA policy recommendations should be tailored for each environment.

## Background

The health benefits of regular physical activity (PA) have been demonstrated for a number of chronic conditions, ranging from depression to obesity [1]. However, in contrast to biochemical risk factors, such as cholesterol or glucose, or physiologic parameters, like blood pressure, the construct of health-promoting PA is based on a variety of working definitions and is difficult to measure. In the chronic disease literature and in most public health campaigns in middle- and upper-income countries, PA is virtually synonymous with voluntary, leisure-time exercise undertaken to induce cardio-respiratory fitness [2]. Historically, nutritionists have been particularly interested in PA as a component of total energy expenditure which can then be used to calculate the number of calories required to maintain healthy body mass [3, 4]. To a lesser degree, surveys with structured questionnaires have attempted to capture how PA is built into an individual’s occupation, mode of transport and leisure time activities [5] and these have begun to focus on populations living in low-income countries, where the construct of PA is distinctly different from developed countries. 80% of the world’s populations live in middle- or lower-income countries, which also carry 80% of the global burden of non-communicable diseases [6] and yet are disproportionately under studied.

This difference in how populations living in low-, middle or upper-income countries accumulate daily PA, i.e. patterns and intensity, is an important part in addressing the global PA movement, particularly when it comes to international public health messaging [7–9]. It’s becoming clear that a “one-size-fits-all” message may not be appropriate and that there is likely a trade-off between occupational and leisure time PA between the different settings [10]. From an international perspective the emphasis placed on accumulating PA from purposive walking or through use of a gym or health club will not be appropriate for most of the world’s population [11].

What is currently missing from the literature is an international comparison using objectively measured PA, in combination with self report, to elucidate the construct of PA in each setting. The advent of objective measurement tools that can be used in free-living populations has demonstrated significant limitations of questionnaires and focused interest on all forms of PA, rather than just sports or other activity that is undertaken to induce fitness [12].

We have undertaken an international collaborative study to assess the role of energy expenditure on risk of obesity [13]. The primary hypothesis being tested is whether habitual levels of PA are associated with obesity in countries spanning the epidemiologic transition, using both objective and self-reported PA. The study samples were drawn from an urban, industrialized community, working class neighborhoods in middle income countries, and rural Africa, thus encompassing a broad spectrum of lifestyles, and levels of social development. In this paper we report PA intensity-duration patterns, as measured by accelerometery and self report [14], over a 7 day period. Our aim was to examine differences and similarities in PA intensity/duration and by domain of PA, between countries spanning the epidemiologic transition. These data provide a framework within which the construct of PA can be broadened to include the range of culture-specific patterns observed across most contemporary societies.

## Methods

### Sampling design and participant recruitment

Twenty-five hundred adults, ages 25–45 years, were enrolled in METS between January 2010 and December 2011. A detailed description of the METS protocol has been previously published [13]. In brief; METS enrolled five hundred participants, approximately 50% female, in each of five study sites: rural Ghana, urban South Africa, the Seychelles, urban Jamaica and metropolitan Chicago. These sites were selected as they represent a broad range of socio economic development, as defined by the UN Human Development Index (HDI), i.e. Ghana is a low HDI country, South Africa is a middle, Jamaica and the Seychelles as high, and the US as a very high HDI country [15]. Each site used their own recruitment strategy; in Ghana, a simple random sample was generated for the age-range of the study from the population census for the rural town of Nkwantakese. In Seychelles, sex- and age-stratified random samples were generated from their respective national censuses. In South Africa, the sex- and age-stratified random sample was drawn from previously enumerated areas of Khayelitsha, the third largest township in the country and adjacent to the city of Cape Town. In Kingston, Jamaica, districts were randomly sampled; beginning from a fixed point in each district (e.g., the north-west corner), and door-to-door recruitment was then carried out. Similarly, in Maywood, IL, USA, all city blocks in the community were randomized and door-to-door recruitment was conducted.

Individuals were excluded if they were diagnosed with infectious disease (e.g. HIV-positive), were pregnant or lactating, or were unable to participate in normal physical activities.

METS was approved by the Institutional Review Board of Loyola University Chicago, IL, USA; the Committee on Human Research Publication and Ethics of Kwame Nkrumah University of Science and Technology, Kumasi, Ghana; the Health Science Faculty Research Ethics Committee of the University of Cape Town, South Africa; the Board for Ethics and Clinical Research of the University of Lausanne, Switzerland; the Ethics Committee of the University of the West Indies, Kingston, Jamaica; and the Health Sciences Institutional Review Board of the University of Wisconsin, Madison, WI, USA. Written informed consent was obtained from all participants.

### Measurements

All measurements were performed early in the morning at outpatient clinics or testing sites, located in the communities.

### Anthropometrics and body composition

Weight was measured to the nearest 0.1 kg using the same model standard calibrated balance at all 5 sites (Seca 770, Hamburg, Germany). Height was measured to the nearest 0.1 cm using a stadiometer (e.g. Invicta Stadiometer, Invicta, London, UK) with the participant’s head held in the Frankfort plane. We used weight and height to calculate BMI (kg/m^2^) and classified participants as normal weight (<25 kg/m^2^), overweight (> = 25- and <30 kg/m^2^) and obese (> = 30 kg/m^2^), and in accordance with international standards [16].

We estimated body composition using bioelectrical impedance analysis (BIA) with the use of a single-frequency (50 kHz) impedance analyzer (model BIA 101Q; RJL Systems, Clinton Township, MI). Fat-free mass (FFM) and fat mass (FM) were estimated from measured resistance by using an equation previously validated in African-origin populations [17].

### Physical activity measurements

#### Physical activity monitoring

Physical activity (PA) was measured using an accelerometer (Actical, Phillips Respironics, Bend, OR, USA). Each participant was asked to wear the accelerometer at all times over 8 days, including during sleep; the only time the monitor should be removed was while bathing, showering, or swimming. From the 8 days, we obtained six complete days of activity from each participant (i.e., a total of eight days after discounting the first and last partially monitored days). We demonstrated a good level of reliability using this technique (83-92%) across all 5 sites, and in keeping with current literature [18]. Currently, there are no universally applied conventions regarding the definition of sleep-time vs. awake-time for continuously collected, i.e. 24-hours, accelerometry data, therefore we assessed activity conducted between the hours of 7 am and 11 pm daily for all sites.

For data analysis, raw data downloaded from the accelerometers were first passed through a SAS macro program designed to infer non-wear time from 90 or more minutes of continuous zero activity counts. This criterion was based on visual inspection of the wear/non-wear patterns across a range of different string-length criteria in a subset of files from each country. A valid day of physical activity monitoring was defined as one having 10 or more hours of wear time, i.e. ≥62% of maximal available wear time. Participant files were included for analysis if they contained four or more valid days, i.e. ≥75% of maximum number of days. Sedentary, moderate and vigorous activity levels were defined using published cut-points: sedentary <100 counts per minute (cpm), moderate 1535–3959 cpm and vigorous ≥3960 cpm [19, 20]. Using the same protocol employed by the National Center for Health Statistics for the analysis of accelerometry data in the continuous National Health and Nutrition Examination Survey [13], minutes defined as comprising sedentary, moderate, vigorous or moderate plus vigorous activity are presented as the total time in minutes accumulated in either 1- or 10- minute intervals. The 10-minute interval may be considered a modified 10-minute bout as, following prior conventions, we allowed for up to 2 minutes of below threshold count activity before considering the bout to be ended [13]. Data are also presented as total activity counts divided by total wear time as an overall measure of average physical activity intensity. We also included average counts and time 1-minute bouts of moderate-to-vigorous activity (MVPA) and sedentary time. Out of 2,506 participants, we excluded 207 participants with insufficient accelerometer data. For the PA patterns analysis we defined weekday as Monday through Friday and weekend day as Saturday and Sunday.

#### Self report physical activity

All participants had self-report PA also assessed using the Global Physical Activity Questionnaire (GPAQ, version 2) [15]. The GPAQ was developed by the World Health Organization (WHO) as part of the WHO STEPwise approach to chronic disease risk-factor surveillance [21] to produce reliable and valid estimates of PA for use in developing countries. The GPAQ is used to estimate the total weekly volume (MET/min) of MVPA in three domains of PA: occupation, travel and recreation and for at least 10 minutes at a time (sloan Ra 2013). These are used to classify persons according to volume of intensity, i.e. low (<600 MET/min), moderate (600–1,499 MET/min), and high (1,500 MET/min vigorous intensity or >3,000 MET/min MVPA). A detailed description of the calculation and categorization is at http://www.who.int/chp/steps/resources/GPAQ_Analysis_Guide.pdf [22].

### Questionnaires

We obtained a basic health history, with a focus on obesity, cardiovascular conditions and diabetes. We further assessed individual occupation using an occupation questionnaire from the U.K. National Statistics Socio-economic Classification (NS-SEC), 2000 edition [23]. Initially, we coded for occupation and industry, then created an indicator variable for manual vs. non-manual class. Occupations coded as non-manual class included: senior, middle, and junior managers; traditional professional (e.g., dentist and lawyer) and modern professional (e.g., teacher, social worker) occupations; and clerical and intermediate occupations (secretary, call center agent). Occupations coded as manual class included technical and craft (e.g., auto mechanic); semi-routine (e.g., postal worker) and routine (e.g., laborer, driver, waiter) manual and service; and farming, agriculture, and fishing occupations. Long-term unemployed (i.e., those not working in the past year) were coded as a separate occupation category.

Data management is centralized at the coordinating center at Loyola University Chicago. All data forms and questionnaires were scanned at each study site and, along with electronic Actical data files, were sent via secure (Bitvise Tunnelier [24]) to the data manager at the coordinating center.

### Statistical analysis

Descriptive statistics including mean levels and distributions were used to summarize the characteristics of participants in each of the five study sites. For continuous measures, we calculated means and standard deviations (e.g. age, weight, % body fat, BMI, minutes of PA and activity counts), and proportions for categorical variables (overweight/obese, female gender, manual labor). These calculations were done by gender and site.

## Results

Sample characteristics are presented in Table 1 (men) and Table 2 (women). Men living in low or middle HDI countries (i.e. Ghana, South Africa) had lower BMI (22 kg/m^2^) than men in middle and high HDI countries (Jamaica, Seychelles and the US). Among the women, this relationship did not exist while women from the US had the highest mean BMI (34 kg/m^2^), women from South Africa had mean BMI of 32 kg/m^2^, and no countries had mean BMI less than 25.0 kg/m^2^. The prevalences of overweight plus obesity (BMI ≥25 kg/m^2^) ranged widely, e.g. 16% of the men in Ghana up to 71% in the US men and 50% of the Ghanaian women up to 84% of US women.

**Table 1:** **Participant characteristics from 5 sites – mean ± SD (men)**

	Ghana	South Africa	Jamaica	Seychelles	United States
Sample size	207	236	249	230	245
Age (y)	34.6 ± 6.7	33.7 ± 5.6	34.0 ± 5.9	36.5 ± 5.1	35.6 ± 6.2
Weight (kg)	63.6 ± 9.1	65.6 ± 13.6	73.1 ± 15.0	80.1 ± 16.0	92.8 ± 24.8
Height (cm)	169.0 ± 6.6	170.9 ± 6.3	176.0 ± 6.7	173.9 ± 6.2	176.6 ± 6.6
Body Mass Index (kg/m^2^)	22.2 ± 2.7	22.4 ± 4.3	23.6 ± 4.5	26.5 ± 4.9	29.7 ± 7.5
Waist Circumference (cm)	77.1 ± 10.5	80.9 ± 11.5	80.3 ± 12.1	89.4 ± 11.8	97.1 ± 21.5
Hip Circumference (cm)	91.8 ± 10.9	94.6 ± 8.4	95.1 ± 9.3	102.8 ± 9.6	109.2 ± 15.9
Fat-free Mass (kg)	53.0 ± 5.1	50.6 ± 6.3	57.5 ± 6.9	59.6 ± 7.7	63.3 ± 9.7
Fat Mass (kg)	10.6 ± 5.3	15.0 ± 8.3	15.7 ± 9.2	20.6 ± 10.0	29.5 ± 16.6
Body Fat (%)	16.0 ± 6.0	21.7 ± 6.9	20.1 ± 7.4	24.5 ± 7.8	29.7 ± 9.0
Systolic Blood Pressure (mmHg)	118.9 ± 13.1	129.0 ± 17.1	121.5 ± 12.8	122.7 ± 14.6	127.9 ± 14.5
Diastolic Blood pressure (mmHg)	68.5 ± 11.4	79.6 ± 13.2	71.2 ± 11.1	75.0 v 11.4	81.0 ± 12.1
Education (y)	9.2 ± 3.8	9.5 ± 2.6	10.6 ± 2.1	13.1 ± 2.4	12.7 ± 1.6
Employed (%)	98.6	91.4	90.8	97.8	85.2
Manual Laborer (%)	60.9	90.4	61.8	54.4	67.1

**Table 2:** **Participant characteristics from 5 sites – mean ± SD (women)**

	Ghana	South Africa	Jamaica	Seychelles	United States
Sample size	293	268	251	270	257
Age (y)	34.0 ± 6.6	33.1 ± 6.0	34.7 ± 6.2	35.8 ± 6.0	35.0 ± 6.3
Weight (kg)	63.6 ± 13.1	82.0 ± 22.3	78.5 ± 18.6	72.1 ± 17.3	91.7 ± 24.4
Height (cm)	158.0 ± 5.7	160.1 ± 6.3	163.2 ± 6.6	161.4 ± 6.5	164.0 ± 6.2
Body Mass Index (kg/m^2^)	25.5 ± 5.2	31.9 ± 8.2	29.5 ± 6.7	27.6 ± 6.2	34.1 ± 8.8
Waist Circumference (cm)	84.2 ± 12.5	96.9 ± 16.6	92.0 ± 13.8	87.9 ± 12.4	101.9 ± 19.6
Hip Circumference (cm)	100.2 ± 13.2	114.2 ± 15.6	107.9 ± 11.6	104.4 ± 12.4	117.0 ± 16.0
Fat-free Mass (kg)	40.8 ± 5.3	45.2 ± 8.0	46.5 ± 7.1	44.7 ± 14.9	49.6 ± 8.6
Fat Mass (kg)	22.8 ± 8.5	36.8 ± 14.8	32.1 ± 12.1	28.1 ± 11.2	42.3 ± 16.4
Body Fat (%)	34.8 ± 6.1	43.4 ± 6.6	39.7 ± 6.1	37.8 ± 6.7	44.6 ± 6.5
Systolic Blood Pressure (mmHg)	110.5 ± 15.2	118.2 ± 18.6	115.2 ± 14.7	110.8 ± 12.8	117.4 ± 16.2
Diastolic Blood pressure (mmHg)	66.2 ± 11.4	76.3 ± 11.8	72.1 ± 11.4	71.2 ± 9.9	79.6 ± 13.2
Education (y)	7.5 ± 4.1	10.0 ± 2.1	10.7 ± 2.0	13.0 ± ±2.5	13.8 ± 2.6
Employed (%)	89.4	81.7	72.1	95.6	83.7
Manual Laborer (%)	90.4	89.3	68.8	26.4	28.9

### Manual labor by site

In South Africa, 91% of the men reported that they engaged in manual labor, compared to 67% of the men in the US sample, and 60% for both Ghana & Jamaica and 54% for the Seychelles. Among the women, 90% of the women from South Africa and Ghana engaged in manual labor, compared to 68% in Jamaica, and 28 and 29% for US and Seychelles.

### Physical activity by accelerometer

Physical activity data by week and weekend is presented as combined men and women (Table 3) and separately for men (Table 4) and women (Table 5). Overall, 93% of the METS participants provided complete accelerometry data as defined by having valid wear time for at least 10 hours per day on a minimum of 4 days of the measurement period. Average accumulated time in vigorous activity were low across sites with peak values of 8 min/d in South African men and hardly any time accumulated at this intensity in women. Among the men, mean MVPA (1-min bouts) was highest in the South African men (55.8 ± 34.7 min/d), and lowest amongst the Jamaican men (30.0 ± 23.7). South African men also accumulated the greatest amount of time in 10-min bouts of MVPA (32 min/day). The mean activity intensity was significantly lower among the women and ranged from 15.4 ± 17.8 min/d in US women to 26.0 ± 16.8 min/d among the Ghanaian women. Furthermore, women in Ghana, South Africa and Seychelles only managed to accumulate one 10-min bout in MPVA, while US and Jamaican women were unable to register a single 10-min bout. However, when the data are summarized in 1 minute MVPA bouts, about half of the men (56%) and 25% of the women managed to accumulate at least 30 minutes of daily MVPA.

**Table 3:** **Physical activity (accelerometry) for the week and weekend, by site (total)**

	Ghana		South Africa		Jamaica		Seychelles		USA	
	Weekday	Weekend	Weekday	Weekend	Weekday	Weekend	Weekday	Weekend	Weekday	Weekend
# Individuals with Valid Data	451	443	493	493	445	435	444	402	466	450
Mean Wear Time (hr/d)	15.4 ± 0.8	15.9 ± 1.2	16.1 ± 0.9	16.0 ± 1.1	15.3 ± 1.0	15.5 ± 1.6	14.1 ± 1.4	14.0 ± 1.9	15.3 ± 1.0	15.3 ± 1.6
Mean Activity Counts (ct/min)	215.6 ± 86.6	208.1 ± 102.8	196.9 ± 130.3	199.0 ± 144.2	171.4 ± 88.7	154.3 ± 99.4	220.4 ± 111.8	180.6 ± 114.9	179.4 ± 128.9	158.3 ± 125.0
Moderate (min/d in 1-min bouts)	33.4 ± 22.0	32.6 ± 26.5	33.3 ± 27.1	33.1 ± 28.5	24.3 ± 19.7	20.1 ± 21.4	29.4 ± 19.9	18.9 ± 19.5	22.6 ± 26.9	18.3 ± 25.7
Vigorous (min/d in 1-min bouts)	1.3 ± 2.9	1.6 ± 4.2	4.8 ± 11.5	3.9 ± 14.8	1.7 ± 4.6	1.4 ± 4.8	3.1 ± 6.3	2.1 ± 7.4	3.1 ± 8.7	2.6 ± 8.7
MVPA* (min/d in 1-min bouts)	34.7 ± 23.4	34.2 ± 28.8	38.1 ± 33.8	37.0 ± 34.8	26.0 ± 21.7	21.4 ± 23.8	32.5 ± 23.2	21.0 ± 23.4	25.8 ± 31.9	20.9 ± 30.6
Sedentary (min/d in 1-min bouts)	193.7 ± 42.5	202.0 ± 49.2	213.7 ± 47.6	216.7 ± 52.7	217.8 ± 51.6	224.3 ± 62.7	192.2 ± 52.0	194.7 ± 58.6	208.3 ± 47.9	201.8 ± 54.1
Moderate (min/d in 10-min bouts)	13.9 ± 14.4	12.5 ± 16.8	15.9 ± 19.5	13.2 ± 18.0	8.8 ± 12.1	6.6 ± 13.5	11.1 ± 12.3	6.8 ± 12.4	10.2 ± 20.4	8.1 ± 19.3
Vigorous (min/d in 10-min bouts)	0.5 ± 1.9	0.4 ± 2.0	2.7 ± 9.6	1.8 ± 13.5	0.7 ± 3.4	0.4 ± 3.3	1.2 ± 4.1	1.0 ± 5.9	1.7 ± 7.0	1.5 ± 7.5
MVPA (min/d in 10-min bouts)	15.4 ± 15.7	14.6 ± 20.2	21.6 ± 26.8	17.5 ± 25.5	10.8 ± 14.4	8.3 ± 16.8	14.9 ± 16.0	9.6 ± 17.7	14.1 ± 26.9	11.2 ± 25.2
Sedentary (min/d in 10-min bouts)	45.6 ± 34.3	48.4 ± 42.7	54.5 ± 37.2	53.3 ± 43.3	60.9 ± 43.9	68.5 ± 57.6	55.8 ± 43.8	58.9 ± 49.5	48.5 ± 33.9	45.0 ± 37.7

**Table 4:** **Physical activity monitor measures by site (men)**

	Ghana		South Africa		Jamaica		Seychelles		USA	
	Weekday	Weekend	Weekday	Weekend	Weekday	Weekend	Weekday	Weekend	Weekday	Weekend
	182	179	229	229	216	211	211	191	228	225
Mean Wear Time (hr/d)	15.4 ± 0.8	16.0 ± 1.0	16.2 ± 0.8	16.0 ± 1.1	15.2 ± 1.0	15.6 ± 1.6	14.1 ± 1.3	14.1 ± 1.8	15.3 ± 0.9	15.4 ± 1.6
Mean Activity Counts (ct/min)	252.5 ± 92.1	247.9 ± 122.5	260.5 ± 154.0	261.8 ± 176.2	192.8 ± 102.2	172.3 ± 120.4	256.2 ± 129.0	215.0 ± 140.3	215.2 ± 156.2	186.2 ± 142.3
Moderate (min/d in 1-min bouts)	44.2 ± 23.1	44.6 ± 30.7	47.4 ± 30.1	46.5 ± 31.2	28.3 ± 22.0	24.7 ± 24.8	33.4 ± 22.4	25.5 ± 22.7	30.4 ± 31.5	24.9 ± 29.4
Vigorous (min/d in 1-min bouts)	2.7 ± 4.1	3.3 ± 5.9	9.2 ± 15.6	7.8 ± 20.9	2.9 ± 5.9	2.4 ± 6.5	5.3 ± 8.2	3.9 ± 10.3	5.1 ± 11.7	3.6 ± 10.6
MVPA* (min/d in 1-min bouts)	46.9 ± 24.8	47.9 ± 34.0	56.6 ± 38.2	54.3 ± 39.8	31.1 ± 24.7	27.1 ± 28.0	38.8 ± 27.0	29.4 ± 28.1	35.5 ± 38.4	28.5 ± 35.5
Sedentary (min/d in 1-min bouts)	197.9 ± 46.1	201.1 ± 51.7	207.6 ± 48.7	209.0 ± 53.4	227.6 ± 54.4	229.7 ± 68.7	197.9 ± 53.8	198.3 ± 62.3	207.5 ± 47.8	203.0 ± 50.4
Moderate (min/d in 10-min bouts)	18.9 ± 16.1	17.2 ± 19.7	23.1 ± 23.9	18.3 ± 21.4	9.8 ± 14.3	7.9 ± 16.0	11.2 ± 13.1	9.3 ± 15.0	14.7 ± 24.2	12.5 ± 22.9
Vigorous (min/d in 10-min bouts)	1.1 ± 2.8	0.9 ± 2.9	5.3 ± 13.5	3.6 ± 19.7	1.2 ± 4.4	0.8 ± 4.6	2.2 ± 5.5	1.9 ± 8.4	2.8 ± 9.7	2.0 ± 8.9
MVPA (min/d in 10-min bouts)	22.1 ± 17.9	21.9 ± 25.1	34.4 ± 32.5	26.7 ± 31.9	13.3 ± 17.4	11.0 ± 20.7	17.7 ± 18.5	14.4 ± 22.5	21.0 ± 33.2	16.9 ± 30.4
Sedentary (min/d in 10-min bouts)	51.1 ± 42.4	47.8 ± 44.2	50.8 ± 36.7	45.5 ± 39.5	70.9 ± 48.8	74.9 ± 65.5	62.3 ± 49.2	63.5 ± 51.8	49.9 ± 34.6	44.9 ± 35.3

**Table 5:** **Physical activity monitor measures by site (women)**

	Ghana		South Africa		Jamaica		Seychelles		USA	
	Weekday	Weekend	Weekday	Weekend	Weekday	Weekend	Weekday	Weekend	Weekday	Weekend
# Individuals with Valid Data	269	264	264	264	229	224	233	211	238	225
Mean Wear Time (hr/d)	15.3 ± 0.9	15.9 ± 1.3	16.1 ± 0.9	16.1 ± 1.1	15.3 ± 1.0	15.4 ± 1.6	14.1 ± 1.4	13.9 ± 1.9	15.3 ± 1.0	15.1 ± 1.6
Mean Counts (ct/min)	190.7 ± 72.9	181.2 ± 76.2	141.8 ± 68.1	144.5 ± 74.5	151.2 ± 68.0	137.4 ± 70.6	188.0 ± 81.1	149.5 ± 73.0	145.1 ± 82.7	130.3 ± 97.4
Moderate (min/d in 1-min bouts)	26.1 ± 17.9	24.4 ± 19.6	21.1 ± 16.2	21.5 ± 19.4	20.6 ± 16.4	15.7 ± 16.7	25.7 ± 16.4	12.9 ± 13.5	15.2 ± 18.8	11.8 ± 19.5
Vigorous (min/d in 1-min bouts)	0.4 ± 0.9	0.4 ± 1.4	1.0 ± 2.8	0.6 ± 2.1	0.6 ± 2.5	0.4 ± 1.9	1.1 ± 2.6	0.5 ± 1.3	1.2 ± 3.1	1.6 ± 6.0
MVPA* (min/d in 1-min bouts)	26.5 ± 18.3	24.9 ± 20.0	22.1 ± 17.6	22.1 ± 20.1	21.2 ± 17.1	16.1 ± 17.4	26.8 ± 17.4	13.4 ± 14.3	16.4 ± 20.2	13.4 ± 22.4
Sedentary (min/d in 1-min bouts)	190.9 ± 39.6	202.6 ± 47.5	219.0 ± 45.9	223.4 ± 51.2	208.6 ± 47.0	219.3 ± 56.2	187.1 ± 49.9	191.4 ± 54.9	209.1 ± 48.0	200.5 ± 57.5
Moderate (min/d in 10-min bouts)	10.5 ± 11.9	9.4 ± 13.7	9.6 ± 11.5	8.9 ± 13.1	7.8 ± 9.3	5.5 ± 10.5	11.0 ± 11.5	4.6 ± 8.9	5.9 ± 14.8	3.8 ± 13.6
Vigorous (min/d in 10-min bouts)	0.1 ± 0.5	0.1 ± 1.0	0.4 ± 1.8	0.1 ± 0.9	0.3 ± 2.0	0.1 ± 0.9	0.3 ± 1.6	0.1 ± 1.0	0.6 ± 2.2	1.0 ± 5.7
MVPA (min/d in 10-min bouts)	10.9 ± 12.2	9.6 ± 14.0	10.6 ± 12.9	9.6 ± 14.1	8.5 ± 10.4	5.8 ± 11.5	12.4 ± 12.7	5.2 ± 10.1	7.5 ± 16.5	5.6 ± 16.7
Sedentary (min/d in 10-min bouts)	41.9 ± 26.8	48.8 ± 41.8	57.6 ± 37.4	60.1 ± 45.3	51.4 ± 36.4	62.5 ± 48.4	49.8 ± 37.5	54.8 ± 47.0	47.1 ± 33.2	45.2 ± 40.0

### Weekly physical activity patterns

**Figure 1:**
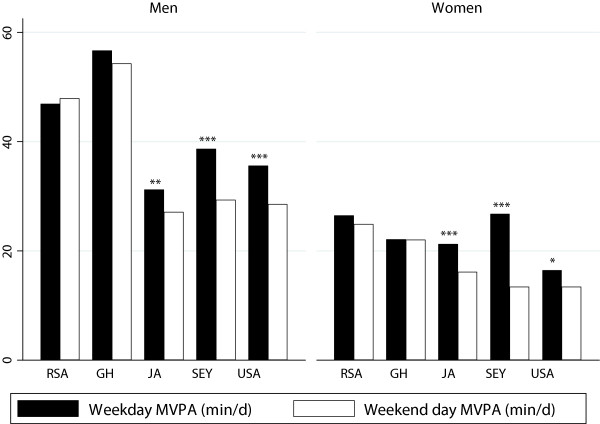
**Accelerometer measured MVPA (min/d) on week and weekend days for men and women, by site.**

Figure 1 presents the week versus weekend MVPA (min/d) patterns for men and women. We found that site differences remained for both men and women comparing week versus weekend, MPVA (1-min bouts), i.e. Monday-Friday versus Saturday and Sunday. South African men accumulated significantly more weekday and weekend day MVPA (p < 0.001) than men in Ghana, who in turn accumulated significantly more MVPA than men in the US, Jamaica or Seychelles. Amongst the women we found that Ghanaian (26.5 ± 18.3 min/d) and Seychellois (26.8 ± 17.4 min/d) women accumulated significantly more weekday 1-min MPVA than women in the US (16.4 ± 20.2 min/d), South Africa (22.1 ± 17.6 min/d) and Jamaica (21.2 ± 17.1 min/d).

Within sites we found significant differences in the amount of daily MVPA accumulated on week versus weekend days. The relationships within sites was however not consistent, for example men in the US, Jamaica and Seychelles accumulated significantly more MPVA (1-min bouts) during the week compared to the weekend, while men in South Africa and Ghana accumulated similar amounts of MPVA, irrespective of whether it was a week or weekend day. This might suggest real international differences in how we separate out weekly PA into occupational and leisure time PA. Amongst the women, it was only women in Jamaica and Seychelles who accumulated significantly different amounts of MPVA on the different segments of the week. These site discrepancies are highlighted when we explore what proportions of each population are meeting PA recommendations on both week and weekend days. For example, only 24% of men in the US, 27% of Jamaicans, 32% of Seychellois accumulated 30 min/d on both week and weekend days, compared to 58% of South African men, and 56% of Ghanaian men. Whereas among the women, only a small proportion of each population met PA recommendations on both types of day. Of note is that twice as many Ghanaian women (20%) met the PA recommendation on both week and weekend days, as US women (8%), Seychellois women (9%) and Jamaican women (12%). 15% Of South African women met the guideline on both types of day.

**Figure 2:**
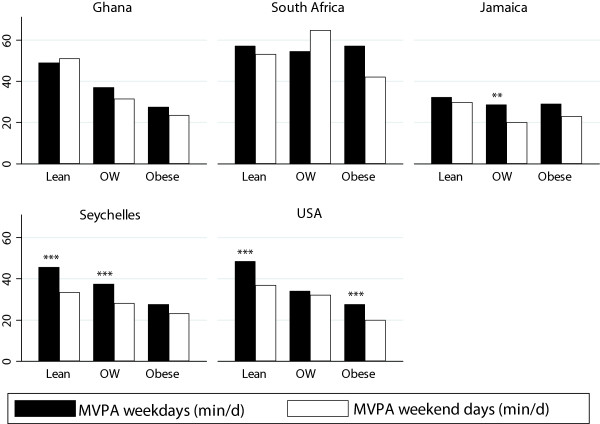
**Accelerometer measured MVPA (min/d) on week and weekend days by adiposity for men, by site.**

**Figure 3:**
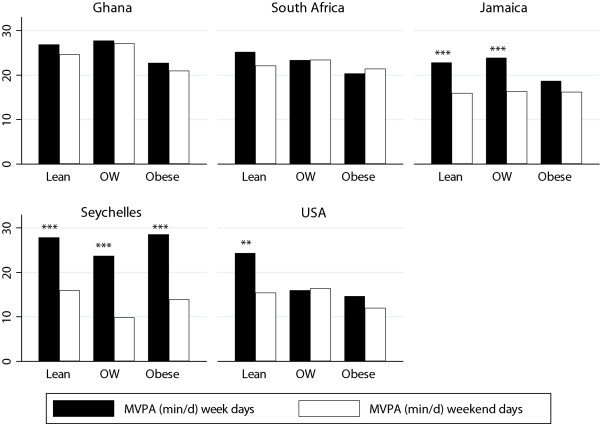
**Accelerometer measured MVPA (min/d) on week and weekend days by adiposity for women, by site.**

In order to explore these differences in greater detail we reanalyzed the data according to level of obesity (Tables 6 and 7, Figures 2 and 3), i.e. lean (<25 kg/m^2^), overweight (25–29 kg/m^2^) and obese (> = 30 kg/m^2^). Among our men, we found lean individuals accumulated significantly more 1-min MPVA (46 min/d) during the week, compared to overweight (37 min/d) and obese individuals (30 min/d) and similarly on weekends (43, 33 & 23 min/d, lean, overweight and obese respectively). This association remained for the women, only after combining overweight and obese women, where lean women accumulated significantly more 1-min MVPA on weekends (25 vs. 21 min/d), and only 2 more minutes on the weekend (21 vs. 23 min/d, NS). These obesity differences remained after adjusting for site adiposity differences.

**Table 6:** **Physical activity monitor measures by BMI group and participation in manual labor (men)**

	BMI < 25		BMI: 25–29.99		BMI > =30		No manual labor		Manual labor	
	Weekday	Weekend	Weekday	Weekend	Weekday	Weekend	Weekday	Weekend	Weekday	Weekend
Mean Wear Time (hr/d)	15.3 ± 1.1	15.6 ± 1.5	15.1 ± 1.3	15.2 ± 1.8	15.0 ± 1.3	15.1 ± 1.9	15.0 ± 1.2	15.3 ± 1.8	15.4 ± 1.2	15.5 ± 1.5
Mean Activity Counts (ct/min)	250.1 ± 139.5	232.0 ± 145.2	222.0 ± 128.0	207.4 ± 164.4	196.4 ± 106.7	167.0 ± 110.0	209.3 ± 105.6	187.4 ± 111.5	249.1 ± 143.2	232.7 ± 154.8
Moderate (min/d in 1-min bouts)	40.7 ± 28.4	38.0 ± 30.6	32.7 ± 25.7	28.7 ± 28.5	26.9 ± 22.7	21.1 ± 23.3	30.8 ± 23.4	27.0 ± 27.2	39.9 ± 28.7	37.2 ± 30.5
Vigorous (min/d in 1-min bouts)	6.1 ± 11.0	4.9 ± 10.0	4.5 ± 11.2	4.5 ± 19.9	2.8 ± 6.1	1.6 ± 4.9	3.5 ± 5.8	2.7 ± 5.6	6.0 ± 12.1	4.9 ± 14.5
MVPA* (min/d in 1-min bouts)	46.7 ± 34.5	42.9 ± 36.0	37.2 ± 30.1	33.1 ± 36.5	29.6 ± 25.6	22.7 ± 25.3	34.3 ± 26.1	29.7 ± 30.0	45.9 ± 35.2	42.1 ± 37.0
Sedentary (min/d in 1-min bouts)	207.6 ± 51.3	209.5 ± 58.0	211.0 ± 50.9	209.2 ± 57.8	205.1 ± 52.5	204.0 ± 62.5	207.8 ± 50.8	212.0 ± 60.5	207.1 ± 51.9	206.7 ± 58.3
Moderate (min/d in 10-min bouts)	17.6 ± 20.8	14.5 ± 19.7	13.6 ± 18.9	13.2 ± 22.1	10.4 ± 15.7	7.2 ± 15.2	11.8 ± 16.1	9.7 ± 17.6	17.7 ± 21.0	15.1 ± 21.0
Vigorous (min/d in 10-min bouts)	3.0 ± 8.8	1.9 ± 7.2	2.5 ± 9.6	2.7 ± 19.4	1.4 ± 4.5	0.7 ± 3.2	1.5 ± 4.0	1.0 ± 3.5	3.1 ± 10.0	2.2 ± 13.1
MVPA (min/d in 10-min bouts)	25.1 ± 28.3	20.4 ± 26.9	19.1 ± 24.0	18.5 ± 31.9	13.7 ± 19.3	9.5 ± 18.1	16.1 ± 19.1	13.3 ± 21.2	25.0 ± 28.9	21.0 ± 29.4
Sedentary Act (min/d in 10-min bouts)	57.6 ± 44.6	55.4 ± 51.6	56.3 ± 40.7	54.9 ± 43.8	55.8 ± 42.5	54.2 ± 50.2	57.8 ± 41.7	59.1 ± 51.0	55.3 ± 43.5	52.6 ± 49.5

**Table 7:** **Physical activity monitor measures by BMI group and participation in manual labor (women)**

	BMI < 25		BMI: 25–29.99		BMI > =30		No manual labor		Manual labor	
	Weekday	Weekend	Weekday	Weekend	Weekday	Weekend	Weekday	Weekend	Weekday	Weekend
# Individuals with Valid Data	394	375	327	317	512	496	403	376	679	668
Mean Wear Time (hr/d)	15.1 ± 1.2	15.3 ± 1.7	15.2 ± 1.3	15.2 ± 1.8	15.4 ± 1.2	15.5 ± 1.6	14.9 ± 1.3	14.7 ± 1.8	15.5 ± 1.1	15.7 ± 1.5
Mean Activity Counts (ct/min)	179.5 ± 81.2	158.8 ± 79.0	168.8 ± 77.4	156.0 ± 86.5	148.0 ± 72.0	138.4 ± 76.9	156.6 ± 75.9	137.5 ± 85.8	167.2 ± 77.7	156.3 ± 77.7
Moderate (min/d in 1-min bouts)	24.7 ± 17.4	19.0 ± 17.6	23.0 ± 17.9	18.7 ± 19.9	18.8 ± 17.2	16.0 ± 18.5	19.9 ± 16.4	13.5 ± 17.8	22.9 ± 18.2	20.1 ± 19.0
Vigorous (min/d in 1-min bouts)	1.2 ± 2.9	0.9 ± 2.8	0.7 ± 1.8	0.6 ± 2.6	0.7 ± 2.5	0.6 ± 3.5	1.1 ± 2.9	1.0 ± 4.6	0.7 ± 2.1	0.5 ± 2.0
MVPA* (min/d in 1-min bouts)	25.9 ± 18.6	19.9 ± 18.9	23.6 ± 18.4	19.3 ± 20.9	19.5 ± 18.0	16.6 ± 19.3	21.0 ± 17.4	14.5 ± 19.6	23.5 ± 19.1	20.6 ± 19.7
Sedentary (min/d in 1-min bouts)	201.0 ± 48.6	209.6 ± 54.0	201.0 ± 43.8	203.3 ± 50.3	205.7 ± 49.0	209.8 ± 57.4	199.7 ± 47.5	202.3 ± 55.9	205.9 ± 45.5	211.6 ± 53.1
Moderate (min/d in 10-min bouts)	10.1 ± 11.6	7.1 ± 11.5	9.3 ± 11.7	6.7 ± 12.5	8.0 ± 12.6	6.2 ± 13.0	7.8 ± 11.1	4.6 ± 11.9	9.7 ± 12.7	7.8 ± 12.8
Vigorous (min/d in 10-min bouts)	0.4 ± 1.9	0.3 ± 1.9	0.2 ± 1.2	0.2 ± 1.8	0.3 ± 1.8	0.3 ± 3.4	0.5 ± 2.2	0.5 ± 4.3	0.2 ± 1.2	0.1 ± 1.2
MVPA (min/d in 10-min bouts)	11.5 ± 13.3	8.0 ± 13.2	10.1 ± 12.4	7.4 ± 13.8	8.8 ± 13.5	6.9 ± 14.0	9.2 ± 12.4	5.7 ± 13.8	10.5 ± 13.7	8.3 ± 13.9
Sedentary Act (min/d in 10-min bouts)	51.4 ± 36.4	56.6 ± 43.6	47.1 ± 31.4	51.3 ± 42.6	49.6 ± 35.3	54.4 ± 47.3	49.0 ± 34.7	54.2 ± 45.4	50.0 ± 33.9	53.7 ± 44.9

### Moderate physical activity

**Figure 4:**
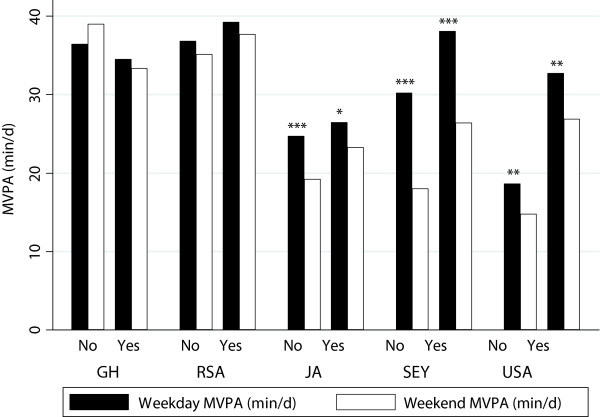
**Accelerometer measured MVPA (min/d) on week and weekend days by manual labor, by site.**

It is apparent that few individuals in our international study engage in purposeful exercise or PA, or accumulate any vigorous PA; therefore we were interested in understanding the patterns of moderate PA. Men accumulated significantly more 1-min bout MPA than women (35.5 ± 25.9 vs. 20.6 ± 15.8 min/d) and that the magnitude of these differences remained when exploring the data for both weekdays (36.6 ± 27.4 vs. 21.8 ± 17.6 min/d) and weekends (33.2 ± 29.8 vs. 17.7 ± 18.7 min/d). As expected; men who reported engaging in manual labor occupations accumulated significantly more MPA than those who reported engaging in non-manual occupations (30.8 ± 23.4 vs. 39.9 ± 28.7 min/d, p < 0.001), with differences persisting on the weekends (27.0 ± 27.2 vs. 37.2 ± 30.5 min/d, p < 0.001) (Table 6, Figure 4). Amongst the women the magnitude of the difference was smaller, yet remained significantly greater for those reported engaging in manual labor (19.9 ± 16.4 vs. 22.9 ± 18.2 min/d, p < 0.001), and similarly to the men, remained significantly different comparing week and weekend days (Table 7). Lean men accumulated significantly more MPA on weekday’s than overweight and obese men (40.7 ± 28.4, 32.7 ± 25.7, and 26.9 ± 22.7 min/d, p < 0.001), while amongst the women differences were much smaller, but remained significant (24.7 ± 17.4, 23.0 ± 17.9 and 18.8 ± 17.2 min/d, p < 0.05) for weekdays. Interestingly, men were 22% less likely to be engaged in manual occupations if they were overweight and obese (p < 0.001), while amongst this women, these differences were smaller and not significant.

### Physical activity by GPAQ

**Figure 5:**
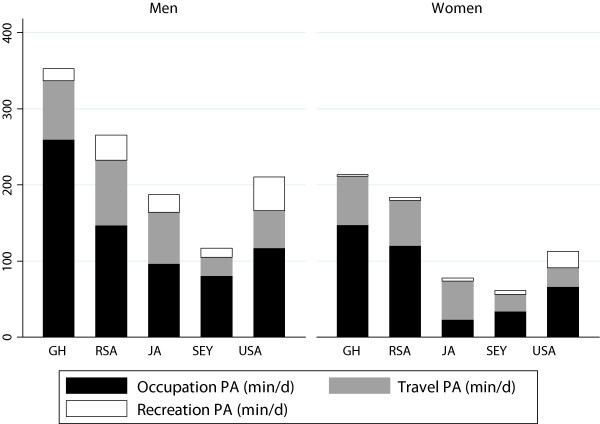
**Self-report PA (min/d, GPAQ) for occupation, travel and recreation PA, by site for men and women.**

The GPAQ captures self-reported PA (MET/min) in 3 different domains: occupational, travel and recreation and 2 different intensities: moderate and vigorous (Tables 8 and 9, Figure 5). These domains are summed to estimate total PA and assigned a PA level according to their volume of activity, low (<600 MET/min), moderate (600–1,499 MET/min), and high (1,500 MET/min vigorous intensity or >3,000 MET/min. Overall, 51.1% of the participants were classified as highly physically active, 25.9% as moderately physically active and 23.0% in low physical activity category. Men reported significantly greater amounts of PA than women, 63.0% of the men were classified as high, compared to 42% of the women (p < 0.001), but more women reported being moderate physically active, 29.4 vs. 22.8% and more women were had low physical activity, 28.4 vs. 14.3%. Over 76% of Ghanaian and South African men were classified as highly physically active compared to only 41% of men from Seychelles. Similarly, the majority of the Ghanaian and South African women were classified as highly physically active, compared to only 28% of women from Seychelles.

**Table 8:** **Physical activity by GPAQ for men, by site (median, inter quartile range)**

	Ghana	South Africa	Jamaica	Seychelles	USA
% high activity	81.3	76.4	54.6	41.0	63.2
% moderate activity	11.0	18.3	23.2	40.1	20.2
% low activity	7.7	5.2	22.2	18.9	16.7
Work daily PA (min)	330.0 (262.5)	120 (170)	147.9 (305.7)	42.9 (154.3)	171.4 (297.5)
Travel daily PA (min)	51.4 (95.7)	51.4 (122.9)	43.9 (100.0)	21.4 (27.9)	30.0 (71.4)
Recreation PA (min)	25.7 (37.1)	85.7 (94.3)	34.3 (60.0)	25.7 (34.3)	35.7 (19.3)
Total daily PA (min)	342.9 (340.7)	240 (312.9)	114.3 (252.9)	72.9 (126.4)	131.4 (285.7)
Sedentary time	120.0 (150)	420.0 (360.0)	300.0 (300.0)	300.0 (180.0)	360.0 (240.0)
Daily moderate Work (min)	128.6 (265.7)	90.0 (145.7)	85.7 (158.6)	42.9 (88.6)	96.4 (171.4)
Daily Vigorous work (min)	214.3 (231.4)	25.7 (60.0)	87.9 (234.3)	85.7 (205.7)	111.4 (201.4)
Daily moderate recreation (min)	23.6 (46.1)	51.4 (68.6)	20.0 (35.7)	17.1 (25.7)	21.4 (38.6)
Daily vigorous recreation (min)	17.1 (21.4)	51.4 (68.6)	34.3 (47.1)	38.6 (42.9)	33.2 (34.3)

**Table 9:** **Physical activity by GPAQ for women, by site (median, inter quartile range)**

	Ghana	South Africa	Jamaica	Seychelles	USA
% high activity	56.9	69.1	44.5	28.3	37.0
% moderate activity	18.2	23.4	34.9	50.6	22.7
% low activity	24.9	7.6	44.5	28.3	40.3
Work daily PA (min)	171.4 (212.1)	111.4 (124.3)	51.4 (157.1)	42.9 (74.3)	55.7 (197.1)
Travel daily PA (min)	42.9 (82.9)	45.0 (68.6)	30.0 (49.3)	21.4 (19.3)	21.4 (51.4)
Recreation PA (min)	12.9 (12.1)	64.3 (102.9)	30.0 (51.4)	17.1 (21.4)	25.7 (37.1)
Total daily PA (min)	175.7 (278.6)	150 (150.0)	39.3 (85.0)	42.9 (64.3)	55.7 (160.0)
Sedentary time	150.0 (150.0)	510.0 (275.0)	300.0 (300.0)	300.0 (300.0)	450.0 (320.0)
Daily moderate Work (min)	128.6 (184.3)	90.0 (88.6)	51.4 (118.6)	42.9 (75.0)	51.4 (162.9)
Daily Vigorous work (min)	124.3 (162.9)	23.6 (42.9)	60.0 (162.9)	38.6 (74.3)	77.1 (187.9)
Daily moderate recreation (min)	11.8 (7.5)	60.7 (68.6)	24.4 (17.1)	17.1 (12.9)	17.1 (24.3)
Daily vigorous recreation (min)	8.6 (6.4)	44.3 (111.4)	31.1 (49.3)	25.7 (17.1)	25.7 (21.4)

Unlike our objective data, the GPAQ is able to provide insight into how respondents accumulate their total daily PA in different domains (i.e. occupational, travel and recreation). Overall the men reported accumulating more work (190 vs. 137 min/d, p < 0.001) and travel PA (74 vs. 59 min/d, p < 0.001), and over 20 more minutes of recreation PA (60 vs. 39 min/d) when compared to women (data not shown). Among the men, Ghanaians reported significantly greater total daily PA (min/d), accumulating on average 100 more minutes than South African men, 145 more minutes than US men, 153 more minutes than Jamaicans and 241 more minutes than Seychellois. Interestingly, among the men, there was no difference for the reported daily occupational PA (min/d), by whether or not respondents were engaged in manual labor. Similar to the men, Ghanaian women reported significantly more total daily PA (min/d), than all other sites, with women from Seychelles accumulating the least amount of total daily PA (66.3 min/d). These differences were primarily driven by participants in the Seychelles reporting less than half the amount of occupational PA. Unlike the men, women who engaged in manual labor, reported over twice the amount of daily occupational PA (165.0 vs. 82.4 min/d), than women not engaged in manual labor.

### Self-reported PA by adiposity

We also explored the respondents reported PA by BMI categories and found that lean men (<25 m/2) reported almost a one hour/day more occupational PA, almost 15 minutes more of travel PA, but similar amounts of recreational PA. Among the women, lean respondents reported similar amounts of occupational and travel PA, but significantly more recreation PA (15 min/d) than overweight and obese women. Even though, this study was not an attempt to compare objective MVPA against self report PA, we did calculate the correlation coefficient between the two methods and found it to be modest (r = 0.26, p < 0.001, N = 2,298). This statistic was however, driven primarily by the men living in Ghana, Seychelles and the USA where these relationships were all significant and coefficients were in excess of 0.2. Among the women, the correlations were all low (<0.15) and only significant in Ghana and Jamaica. Finally, and in contrast to our accelerometry data, over 70% of the men and 65% of the women reported accumulating 30 or more minutes of moderate and vigorous daily PA.

## Discussion

The primary hypothesis being tested is whether habitual levels of PA are associated with obesity in countries spanning the epidemiologic transition, using both objective and self report PA. This combination allows for the assessment of patterns of PA, both by intensity (moderate and vigorous) as well as day of the week (week versus weekend) and finally domains of PA (work, travel and recreation). As expected, the prevalence of obesity was significantly different among our 5 sites, with adults from Ghana presenting with the lowest rates (38% overweight/obese) and US adults with the highest (78% overweight and obese). This is very much in keeping with the current published prevalence rates [25] and confirms that our samples are characteristic of their populations.

Overall, men accumulated almost 20 minutes more a day of objectively measured moderate and vigorous PA (MVPA, in 1-minute bouts) than women, with Ghanaian and South African men accumulating the greatest amount. Notably, the site averages for MVPA among the men were all in excess of the current WHO recommendations [26] for PA, while none of the site averages for the women exceeded 30 min/d for MVPA. Women from Ghana were the closest to the recommended goals, accumulating 26 min/d, while US women accumulated only half of the recommended amount of daily PA (15 min/d). This sex difference in daily PA has previously been reported. For example, Cook et al. (2009) found that rural black South African men accumulated significantly more objectively measured pedometer steps than women. In this study, however, the majority of the participants (66%) were classified as physically active (>10,000 steps/d), with only 8% of these participants were classified sedentary (<5000 steps/d). Our data is however, in line with data published by Hallal et al. [8] reporting that US men accumulate 33 min/d of MVPA and US women around 19 min/d of MVPA.

The lower daily PA among the women from the US, is in keeping with the premise that rural dwellers are more physically active than urban dwellers, or/and that populations in developing countries are more active than populations in developed countries [27–29]. Dumith et al. [29] performed a meta-analysis using self reported PA data collected using the IPAQ instrument, in 76 countries, spanning the Human Development Index (HDI). Physical inactivity was higher amongst women compared to men, with differences being greatest among men and women from low HDI countries. Results from the meta-regression indicated that countries with a greater prevalence of physical inactivity, increased as a function of HDI. Similarly, the impact of urbanization on PA has repeatedly been reported. Both Assah [28] and Cook [27] compared rural and urban African populations and in both instances found that the total PA was higher in the rural populations.

In an effort to understand the HDI differences in PA, we explored week versus weekend MPVA. In our study; >90% of men from South Africa were classified as participating in manual labor, compared to only 54% of men in the Seychelles and it could be expected that MPVA would be significantly different according to the day of the week, i.e. week versus weekend, for South Africans at the very least. However, we found that there was no difference in the amount of MVPA accumulated by the most active men in our study (i.e. Ghana and South Africa, Figure 4. It should be noted here, that this may be an artifact of the instrument itself, since it is known that accelerometry may not be sensitive to any increases in energy expenditure during activities, such as weight-bearing manual labor, walking up stairs or activities in postures requiring significant isometric muscle activities [30–32]. On the other hand, this observation might be real, and in the case of South African and Ghanaian men, at the very least, there may be some continuation of casual manual labor on weekends, and outside of the regular work week. Instead it was the men from the US and Seychelles, who completed between 7–10 minutes less on the weekend days, suggesting that they are accumulate their PA from work and travel.

This pattern persisted for the women, where women from South Africa and Ghana accumulated similar amounts of MPVA, irrespective of the time of the week, while women from the US, Jamaica and Seychelles, accumulated significantly less MVPA on the weekend. This difference was most striking amongst the Seychellois where their weekend MVPA time was reduced by more than 50% (27.9 vs. 13.4 min/d) and be in line with a higher HDI country status, where the work week is traditionally Monday through Friday. Due to the limitations of the GPAQ instrument, we were unable to separate out week versus weekend PA domains, which may have provided insight into differences between upper and low HDI weekly PA patterns. Our results, however, are different from the South African study by Cook [27], where participants accumulated significantly more steps on the weekend days versus week days and these study differences may reflect the different settings. All of the participants in that study were from rural villages, although in our study the participants from Ghana were also from one rural village and here we did not find differences among the different days of the week.

With regards to PA patterns and adiposity, we found that lean participants accumulated significantly more MVPA than overweight and obese participants. This finding has previously been reported in a number of studies [33–36]. Among the men, the difference between lean and obese men was as much as 17 minutes on week days (47 vs. 30 min). The difference was not as marked for the women, where lean women accumulated only 6 minutes more than obese women on week days (25 vs. 19 min, p < 0.001); and is probably a result of the overall lower objectively measured PA among all the women. In the South African study, it was found that obese participants walked on average almost 2000 steps per day less [27]. Previous studies have estimated 2000 steps to be approximately 1 mile per day [37], or approximately 15 minutes of walking according 2009 National Household Travel Survey [38]. This is of similar magnitude to the difference found among the men in the current study (17 min/d). Interestingly however, regardless of BMI status, the magnitude of the difference between groups was similar for week versus weekend days and it is apparent from our data that our participants were not as active on the weekends, at least in Jamaica, the Seychelles and USA, all considered middle and upper HDI countries. This is in contrast to the South African study, which found that participants were significantly more active on Saturdays as a result of increased travel physical activity (walking).

This may have important implications for public health messaging, where current WHO and US Surgeon General PA [26, 39–41] guidelines suggest that adults accumulate 30 minutes of MVPA on most days (5 or more) and in bouts of 10 minutes or more [8, 10]. It is apparent from our objective PA data that, and at least among the men in our study, that most already accumulate 30 minutes of MVPA, at least in 1-minute bouts, verified by objective monitoring and in spite of their BMI status or whether they were engaged in manual labor or not. Even amongst the obese men (>30 kg/m^2^), the mean weekly accumulated MVPA was 30 min, in 1-minute bouts. When the data is analyzed in 10-minute bouts, only South African men meet PA guidelines. While among the women, none of the 3 BMI groups achieved an average of 30 minutes of objectively measured MVPA per day in 1-minute bouts; however, those in the lean and overweight groups did get close to 25 minutes. When using the 10-minute PA guideline, women in Ghana, South Africa and the Seychelles all accumulate ten minutes of daily MVPA. Although the aim of our study want not directed towards a formal validation of the GPAQ, we noted substantial differences in the amount of MVPA between the instruments. This raise questions about the accuracy of the prevalence estimates of the proportion of sufficiently active individuals obtained by the GPAQ instrument from these countries and locations. For example, using self report data, the men in this study all accumulate well over 60 minutes of daily PA, with those in Ghana and South Africa, reporting over 4 hours. The agreement between the GPAQ instrument and objective MVPA in our study, among the men, were all in excess of 0.2 (p < 0.001), indicating a moderate association, whereas the agreement amongst the women were all very low and only significant among women from Ghana, Jamaica and US. Despite this, women from all 5 sites reported accumulating in excess of 30 minutes of PA per day. One of the mainstays of the global public health response to deal with the world-wide obesity epidemic is to increase overall daily PA, to the point of 30 minutes on most days for health [26, 39, 40] and 60 minutes a day to prevent obesity [42]. To be clear, both the WHO and US surgeon general PA guidelines indicate that the accumulation of PA is not restricted to only leisure or recreational time, but may include “leisure time PA, transportation (e.g. walking or cycling), occupational (i.e. work), household chores, play, games, sports or planned exercise, in the context of daily, family, and community activities” [26, 39]. It is apparent, at least from our data comparing 5 different HDI settings, and among the men, that most already accumulate close to or even more than the recommended amount of daily PA and this provides support for the need for either revisiting the dose of PA recommended for health benefits and to focus more public health messaging on energy intake, in addition to PA.

The large discrepancy between objectively measured PA and self report PA in our study is consistent with observations by Cook et al. [27], who in fact found fairly low levels of sedentarism (8%) among South African adults from a rural setting, compared to previously published reports of sedentarism, using self-report data, of between 34-39% among men and women, respectively. This highlights several issues related to trying to capture PA among participants from settings outside of the developed world, particularly for multi-country studies such as ours, which rely on only self report tools (e.g. GPAQ) to assess PA. It may be that respondents are simply better able to report leisure time PA [43].

Finally, this discrepancy highlights the importance of using objectively measured PA; Troiano found that for the US population, approximately 50% of the adults reported meeting the Surgeon general’s guidelines on PA (accumulate moderate of vigorous aerobic activity for at least 30 minutes/day), while the objective data indicated that far less than 5% of the adult population met this guideline [13]. It is clear from our data, that objectively measured and self-reported PA may provide different results. Cook et al. has suggested several reasons for this disconnect; firstly it is not uncommon for the misclassification of sedentary and light activity as moderate activity [27]. Secondly, the time spent being PA may be overestimated, for e.g. the GPAQ probes PA measured in bouts of 10-minutes and it is not unusual for adults to round up their estimated time. In addition, there are activities which are poorly captured by an accelerometer (i.e. weight bearing activities, such as during construction work) which add to the discrepancy between assessment methods but also highlights that not all discrepancy is due to the limitations of self-report; these methods can be seen as complimentary. Nonetheless, if the goal of WHO PA guidelines is to encourage people to get more physically active and intervention efforts were targeted only at inactive individuals as per self-reported activity, we would likely be missing substantial proportions of the population when the majority of adults are already reporting fairly high levels of PA. With this in mind, population-wide efforts to increase physical activity may be a safer strategy.

## Conclusion

While there is some association for PA with obesity, this relationship is inconsistent across the epidemiologic transition and suggests that PA policy recommendations should be tailored for each environment. For example, it is clear from our data that PA patterns among countries at the lower spectrum of the Human Development Index, do not display the variation between week and weekend days, as those countries further along the spectrum. In addition, within sites, we find that men and women display different MVPA patterns, suggesting that a one-size-fit all approach may not be answer to addressing the global physical inactivity epidemic. In 2012, Hallal et al. [9] issued a global challenge to make PA a public health priority, particularly in low-income and middle income countries, where the vast majority of the world’s population carry a disproportionate amount of the global burden of non-communicable disease and yet are greatly unstudied. Our study represents a step in the right direction to addressing this striking imbalance and provides clear evidence that this problem is complex and will require both a global effort and local effort in the coming decade.
